# COVID-19 Pandemic and Its Effect on Resident Physicians’ Mental Well-Being: A Cross-Sectional Study in Kuwait

**DOI:** 10.7759/cureus.33606

**Published:** 2023-01-10

**Authors:** Mariam Ayed, Anwar Yazdani, Hind Esmaelili, Abdulla Alsaleh, Ahmed Sultan, Esam Alamad, Ali Bander, Hanouf Rawdhan

**Affiliations:** 1 Neonatal, Maternity Hospital, Al-Shuwaikh, KWT; 2 Anesthesia and Critical Care, Alsabah Hospital, Sharq, KWT; 3 Anesthesia and Critical Care, Farwaniya Hospital, Farwaniya, KWT; 4 Anesthesia and Critical Care, Amiri Hospital, Safat, KWT; 5 Anesthesia and Critical Care, Al-Adan Hospital, Al Ahmadi, KWT; 6 Anesthesia and Critical Care, Jaber Alahmad Hospital, Hawalli, KWT; 7 Anesthesia and Critical Care, Kuwait Cancer Control Center, Shuwaikh, KWT

**Keywords:** stress and depression, dass-21 scale, covid 19, program’s residents, mental wellbeing

## Abstract

Objective: Concerns about COVID-19's long-term consequences on the mental health of frontline health professionals are mounting as the entire world strives anew to contain it. The primary objective of this research is to describe the impact of working during the COVID-19 pandemic on resident physicians' mental health.

Subject and methods: A cross-sectional online survey using the Google Forms platform was conducted from May 1 to May 30, 2021, on 311 residents currently enrolled in a residency program at the Kuwait Institutional of Medical Specialization (KIMS). Socio-demographic details of each resident physician were collected and the scores related to depression, anxiety, and stress were measured using the previously validated depression anxiety stress scale-21 (DASS-21).

Results: Higher stress and depression scores were seen in those who were devoid of the option to work with COVID-19 patients, who reported that working during the pandemic affected their study schedule, and who lost off-service training time. Further, the anxiety scores were significantly higher in females.

Conclusion: The impact of the ongoing pandemic on residents’ mental health is grave, necessitating psychological treatment and support. The study discovered various factors linked to depression, anxiety, and stress. As a result, these aspects must be regarded to protect the doctors' mental health.

## Introduction

The COVID-19 pandemic evolved from an immediate health emergency to a systemic problem that had far-reaching consequences in people's lives. Because of its progression from a health scare to a global economic and social disaster, the effects of COVID-19 have been unparalleled [1].

Past investigations of the effects of the severe acute respiratory syndrome, influenza, and Ebola epidemics on suffering individuals and healthcare workers (HCW) revealed a significant neuropsychiatric connection [2]. COVID-19's effects on people's mental health and well-being were being explored more [3,4]. Even before the COVID-19 pandemic, a considerable body of research has shown that resident physicians experience burnout, depression, and anxiety while in training [5]. As a result, the possibility of a reduction in their well-being due to the COVID-19 pandemic is very plausible.

Like many other countries, Kuwait's national healthcare system was strained during the COVID-19 epidemic [6]. Even in high-income countries with well-resourced healthcare systems, overburdened healthcare services confronted considerable operational and logistical issues. As a result, many healthcare staff have been deployed due to the pandemic response [7]. Many resident physicians, despite their specializations, were associated with the management of COVID-19 patients in a variety of setups such as quarantine areas, swabbing areas, emergency departments, COVID-19 wards, and ICUs, starting from February 24 (when the first case of COVID-19 was diagnosed in Kuwait) to the end of September 2020. These kinds of scenarios were also documented in a number of other nations. Resident physicians' rotations were reported to be discontinued in the United Kingdom (UK), and many doctors were redeployed to operate in intensive care units [8].

The specific approach to tackling the epidemic incorporates several interconnected ideas, and multiple studies have found that capability outcomes are closely linked to mental health and social consequences [9]. Despite the growing number of studies looking into the influence of COVID-19 on mental health and well-being, data on the pandemic's broader potential impact is still lacking. Hence, this study aimed to describe the participation of resident physicians in Kuwait in the COVID-19 pandemic and its impact on their mental health and professional training.

## Materials and methods

A cross-sectional online survey was created using the Google Forms platform from May 1 to May 30, 2021. Physicians who were currently enrolled in a residency program at the Kuwait Institutional of Medical Specialization (KIMS) were eligible to participate in this study. The study was authorized by Kuwait's Ministry of Health's Ethical Research Committee. The participants gave their consent to participate in the survey by filling out the questionnaire.

Each residency program's director, chief residents, and head of the department received the questionnaire by email or WhatsApp. The participants were then requested to forward the survey invitation to other residents, with a two-week follow-up reminder. Google Forms were configured to only allow a single submission from each participant to prevent multiple submissions from the same individual. The information gathered was automatically placed into a spreadsheet.

Data collection

The sociodemographic details such as age, gender, marital status, and nationality of each participant were collected. In addition, the physicians were questioned about their participation during the pandemic, whether they had the choice to work with COVID-19 patients, whether they had mentor or supervisor assistance, and whether they had received sufficient personal protective equipment (PPE) training.

The dependent variables, such as depression, anxiety, and stress, were measured using the previously validated depression anxiety stress scale-21 (DASS-21) [10]. The DASS-21 is divided into the following subscales, each with seven items: depression, anxiety, and stress. A 4-point Likert scale is used in the DASS-21. The possibilities for responses range from 0= almost always, 1= often, 2= sometimes, and 3= never. The total of all subscale elements determines the subscale score. The cut-offs for the stated divisions can be enumerated as depression (normal/mild = 0-13; moderate = 14-20; severe/extremely severe = 21+), anxiety (normal/mild = 0-9; moderate = 10-14; severe/extremely severe = 15+), and stress (normal/mild = 0-18; moderate = 19-25; severe/extremely severe = 26+).

Statistical analysis

STATA 14 IC was used to analyze the data. Descriptive statistics were expressed as a number and percentage for categorical variables, while the median and interquartile range (IQR) were used for continuous data. The DASS-21 scores were compared to the demographic and survey responses of the individuals using the Mann-Whitney U test for the comparison of two groups or the Kruskal-Wallis test for the comparison of three or more groups. The significant risk factors related to stress, depression, and anxiety were confirmed using backward stepwise linear regression analysis. Variance inflation factor (VIF) statistics were used to test multicollinearity. The analysis was run in separate models to see how the variables, depression, anxiety, and stress (DASS-21), behaved separately in the multivariate model as they were noted to be multicollinear. In regression analysis, the strength of the link was expressed as a beta (ß) coefficient and a 95% confidence interval (95% CI). All tests were two-tailed, with an alpha of 0.05.

## Results

Participants’ characteristics

A total of 311 responders completed the survey and were included in the present study. Around 50.2% were female, and 58% were 25-29 years old. The socio-demographic details of the enrolled participants are presented in Table 1.

**Table 1:** Sociodemographic characteristics of the participants

Variable	Number (%)
Age, years	
25-29	181 (58.2%)
29-33	62 (20%)
33-37	68 (21.8%)
Gender	
Male	155 (49.8%)
Female	156 (50.2%)
Marital status	
Married	158 (50.8%)
Single	148 (47.6%)
Widowed	1 (0.3%)
Divorced	4 (1.3%)
Nationality	
Kuwaiti	234 (75.2%)
Non-Kuwaiti	77 (24.8%)
Specialty	
Dentistry	17 (5.5%)
Radiology	3 (1%)
General surgery	44 (14.1%)
Pediatrics	42 (13.5%)
Anesthesia/intensive care	78 (25.1%)
Emergency medicine	17 (5.5%)
Internal medicine	68 (21.9%)
Family medicine	22 (7.1%)
Obstetrics and Gynecology	9 (2.9%)
Orthopedics	7 (2.2%)
Infectious disease/public health	4 (1.3%)
Postgraduate year (PGY)	
PGY1	77 (24.7%)
PGY2	79 (25.4%)
PGY3	59 (18.9%)
PGY4	53 (17%)
PGY5	43 (13.8%)
Did you have the option to choose to work with COVID-19 patients?	
Yes	98 (33.6%)
NO	194 (66.4%)
How long have you worked with COVID-19 patients?	
One month	37 (12.7%)
Two months	19 (6.5%)
Three months	40 (13.7%)
Four months	65 (22.3%)
More than four months	130 (44.5%)
Work schedule	
≥ 40 hours	40 (12.9%)
35-39 hours	59 (18.9%)
30-35 hours	115 (36.9%)
<30 hours	97 (31.2%)
Your main working place during the pandemic	
Quarantines	28 (9.6%)
COVID-19 clinics	22 (7.5%)
COVID-19 wards	155 (53%)
COVID-19 intensive care units	87 (29.8%)
Training affected by the pandemic	
No	9 (2.9%)
Yes	302 (97.1%)
Working during the pandemic affected your study schedule	
No	16 (5.1%)
Yes	295 (94.9%)
Lost on service time	
No	71 (22.8%)
Yes	240 (77.2%)
Lost off service time	
No	110 (38.3%)
Yes	192 (61.7%)
During your work during the pandemic, did you receive any support from your senior/supervisor/mentor?	
No	63 (20.3%)
Yes	248 (79.7%)
Did you receive supervised training on personal protective equipment?	
No	74 (23.8%)
Yes	237 (76.2%)
On-service time is defined as the time during your training that is spent in a specialty pertaining to your program. Off service, time is the time during your training spent in a specialty NOT pertaining to your program.	

One hundred and ninety-four (62.4%) participants reported that they could not choose to work with COVID-19 patients. Most KIMS residents (97.1% and 94.9%) believed the pandemic affected their training and study schedule. Lost on and off-service training time was reported in 77.2% and 61.7% of the residents, respectively. The majority of the participants (79.7%) indicated they were devoid of getting support from their supervisors/mentors, and 76.2% of participants stated they lacked adequate PPE training.

DASS-21 results

The median DASS-21 score was noted to be 16 (IQR: 10-24) for stress, 12 (IQR:6-24) for depression, and 10 (IQR: 4-18) for anxiety. The observed values inferred that residents had moderate symptoms of depression and anxiety and normal/mild symptoms of stress. Table 2 shows the median (IQR) scores of stress, depression, and anxiety stratified by the participants’ characteristics and responses.

**Table 2:** Analysis of DASS-21 score based on demographic and participants' response

Variables	Stress	P	Depression	P	Anxiety	P
	Median (IQR)		Median (IQR)		Median (IQR)	
Age						
25-29	18 (12-26)	0.01	14 (8-28)	0.01	12 (4-18)	0.33
29-33	15 (12-26)		12 (6-24)		10 (4-18)	
33-37	13 (8-19)		8 (4-17)		8 (3-16)	
Gender						
Male	16 (10-24)	0.04	12 (4-24)		8 (4-14)	0.02
Female	18 (12-26)		14 (6-26)	0.14	12 (6-19)	
Marital status						
Single	18 (12-24)	0.22	14 (6-26)	0.78	12 (4-18)	0.12
Married	16 (12-26)		12 (6-22)		8 (4-16)	
Widowed	14 (14-14)		14 (14-14)		8 (8-8)	
Divorced	5 (0-10)		1 (0-2)		6 (0-12)	
Nationality						
Kuwaiti	17 (12-26)	0.06	14 (6-26)	0.07	12 (4-18)	0.41
Non-Kuwaiti	16 (8-20)		12 (4-20)		10 (4-16)	
Specialty						
Dentistry	20 (10-26)	0.08	13 (6-24)	0.21	14 (8-18)	0.35
Radiology	22 (0-34)		12 (4-24)		14 (0-18)	
General surgery	14 (10-22)		18 (0-28)		8 (2-14)	
Pediatrics	20 (12-26)		12 (4-24)		8 (4-20)	
Anesthesia/ICU	18 (10-26)		16 (8-26)		12 (4-20)	
Emergency medicine	28 (10-26)		16 (10-30)		14 (8-18)	
Internal medicine	16 (12-21)		12 (6-20)		10 (4-14)	
Family medicine	13 (2-20)		9 (0-14)		11 (2-18)	
Obstetrics and gynecology	14 (12-16)		10 (6-12)		6 (6-12)	
Orthopedics	26 (6-30)		22 (6-28)		4 (2-16)	
Infectious disease/public health	31 (30-32)		20 (14-28)		19 (9-20)	
Enrolled in a residency program						
Yes	16 (12-26)	0.35	12 (6-26)	0.93	10 (4-18)	0.78
No	16 (10-24)		14 (6-24)		10 (4-18)	
Postgraduate year (PGY)						
PGY1		0.95	14 (8-26)	0.37	11 (4-18)	0.43
PGY2	17 (12-24)		12 (5-19)		12 (4-15)	
PGY3	16 (11-26)		17 (7-36)		9 (6-20)	
PGY4	16 (13-30)		8 (4-26)		8 (4-22)	
PGY5	16 (10-26)		12 (0-22)		2 (0-6)	
Did you have the option to choose to work with COVID-19 patients?						
Yes	15 (8-20)	0.01	12 (4-18)		8 (2-14)	0.03
No	18 (12-26)		14 (6-26)	0.02	10 (4-18)	
How long you have worked with COVID-19 patients?						
1 month	14 (6-20)	0.08	10 (6-20)	0.79	6 (2-12)	0.81
2 months	14 (6-16)		6 (2-12)		6 (4-10)	
3 months	16 (12-24)		15 (8-20)		14 (7-15)	
4 months	18 (14-28)		14 (4-30)		14 (4-20)	
>4months	17 (12-24)		12 (6-24)		10 (4-16)	
Work hours (weekly)						
≥ 60 hours	24 (16-31)	0.01	21 (14-29)	0.01	15 (7-21)	0.02
45-59 hours	18 (10-24)		12 (6-18)		12 (8-16)	
40-55 hours	17 (11-26)		14 (5-26)		8 (4-16)	
<40 hours	1 (8-22)		10 (4-22)		8 (4-16)	
Your main working place during the pandemic						
Quarantines	11 (6-21)	0.72	12 (3-20)	0.70	12 (2-20)	0.94
COVID-19 clinics	18 (7-27)		13 (6-24)		14(6-18)	
COVID-19 wards						
COVID-19 intensive care units	19 (12-24)		12 (6-24)		10 (4-16)	
Training affected by the pandemic						
No	7 (4-16)	0.04	4 (0-15)	0.06	6 (1-15)	0.03
Yes	20 (12-30)		14 (8-28)		12 (4-18)	
Working during the pandemic affected your study schedule						
No	7 (6-10)	0.02	2 (0-6)	0.02	3 (0-4)	0.38
Yes	20 (14-30)		14 (8-28)		12 (6-18)	
Lost on service time						
No	14 (6-22)	0.01	8 (0-24)	0.01	4 (2-12)	0.003
Yes	24 (16-32)		23 (12-30)		15 (6-22)	
Lost off service time						
No	16 (10-24)	0.05	10 (4-22)	0.01	10 (4-14)	0.14
Yes	22 (14-30)		20 (12-30)		14 (6-20)	
During your work during the pandemic, did you receive any support from your senior/supervisor/mentor?						
No	22 (16-30)	0.01	24 (8-30)	0.01	14 (6-20)	0.58
Yes	14 (8-20)		10 (4-16)		8 (4-14)	
Did you receive supervised training on personal protective equipment?						
No	20 (10-30)		19 (6-28)		13 (6-20)	
Yes	14 (10-22)	0.03	10 (4-18)	0.01	10 (4-14)	0.24
On-service time is defined as the time during your training that is spent in a specialty pertaining to your program. Off service time is the time during your training spent in a specialty NOT pertaining to your program.						

Stress score

DASS-21 scores revealed that the prevalence of mild, moderate, severe, and extremely severe stress among the respondents was 14.6%, 17.5%, 13.7%, and 10.2%, respectively. The stress scores were significantly higher among those aged 25-29 years compared to those aged 33-37 p<0.005). The stress score was significantly higher in females than in males (p=0.04). Respondents who believed that their training and study schedule were affected by the pandemic, who lost on service training time, and who did not have the option to choose to work with COVID-19 patients had significantly higher stress scores. Moreover, those who had weekly work hours of more than 60 hours and those who did not receive mentor/supervisor support and proper PPE training had significantly higher stress scores.

Depression score

The DASS 21 scores inferred that 12.7%, 19.4%, 10.2%, and 20.1% of the respondents were observed to have mild, moderate, severe, and extremely severe depression. The score for depression was significantly higher among those aged 25-29 years than among those aged between 29 and 33 years (p=0.01). Participants who lost on-and-off-service training time had a higher depression score than those who did not lose on-and-off-service training time (p=0.01). Further, respondents who had weekly work hours of more than 60 hours (p=0.01) had higher median depression scores. Depression scores were significantly higher in those with no mentor or supervisor support (p=0.01) and proper PPE training (p=0.01) than in those with mentor or supervisor support and proper PPE training. 

Anxiety score

The proportion of mild, moderate, severe, and extremely severe anxiety was 13.7%, 17.8%, 8.6%, and 21.7%, respectively, in the studied population. The anxiety score was significantly higher among females than males (p=0.02). In addition, those who lost on service training time (p=0.003), those who did not have the option to choose to work with COVID-19 patients (p=0.03), those training affected by the pandemic (p=0.03) and worked more than 60 hours/week (p=0.02) had higher median anxiety scores.

The variables considered for analyzing their effect on depression, anxiety, and stress subscale scores include age, gender, marital status, nationality, course specialty, current program year of enrolled participants, option to choose to work with COVID-19 patients, time period worked with COVID-19 patients, work schedule, main working place during the pandemic, the effect of training and working schedule during the pandemic, loss on and off training time, any support from your senior/supervisor/mentor and receiving supervised training on PPE.

On multivariate linear regression analysis, higher stress scores were seen in those who were devoid of the option to work with COVID-19 patients (β=5.1, 95%CI: 1.2-9; p=0.01), who reported that working during the pandemic affected their study schedule (β=4.8, 95%CI:1.6-8.1; p=0.004), and who lost off service training time (β=2.7, 95%CI: 0.13-5.2; p=0.034).

Similarly, higher depression scores were observed in those who did not have the option to work with COVID-19 patients (β=6.5, 95%CI: 2.1-10.8; p=0.004), who reported that working during the pandemic affected their study schedule (β=5.8, 95%CI: 2.3-9.3; p=0.001), and who lost off service training time (β=3.2, 95%CI: 0.69-5.7; p=0.013).

Further, anxiety scores were significantly higher in females (β=3.1, 95%CI: 0.09-5.2; p=0.04), among those who feel that their training has been affected by the pandemic (β=2.9, 95%CI: 0.04-5.9; p=0.03) and who lost on service training time (β=2.5, 95%CI: 0.11-4.8; p=0.04).

## Discussion

Our study indicated that various factors were independently associated with the mental state of resident physicians. Gender, the option to choose to work with COVID-19 patients, weekly working hours, training affected by the pandemic, loss of training service time, support from supervisor/mentor, and supervised training on PPE were associated with higher stress, depression, and anxiety scores.

The median stress and anxiety scores were significantly higher in females than in males. The observed results are consistent with previous studies, such as the one done by Lai and co-workers [11]. It is hypothesized that females experience higher stress than males because their stress responses are varied. Women have a hormonal system that is radically different from males, causing them to behave more emotionally. Additionally, females may face more stressors since they often have multiple roles to fulfill in their daily lives [12].

In addition, the present study revealed that median scores of stress and depression were significantly higher among those aged 25-29 years and 29-33 years compared to those aged 33-37 years. This could be related to the fact that most mental health problems begin in early adulthood, but young adults rarely receive mental health support. Furthermore, mental health problems are linked to a higher frequency of physical and emotional disorders, worst academic performance, poor sleep quality, and dysfunctional relationships [13].

In our study, there was a strong association between longer working hours and higher DASS-21 score results. Some research studies have linked long work hours to psychological and occupational stress [14,15]. In addition, long working hours have been linked to an increased risk of cardiovascular disease, chronic fatigue, depressive state, anxiety, self-perceived health, and health behavior [16,17].

Varying types of job conditions are assumed to have different psychological effects due to disparities in the working environment, work intensity, burden, and disease risk. The major finding of the current study is that there is a clear link between high and extended work hours with the precipitation of depression, anxiety, and stress in HCW during the pandemic era. The findings are consistent with the ones reported by other researchers [11,18]. Because of the increased demand for healthcare services during the COVID-19 epidemic, many HCW were forced to work harder, longer, or on more irregular schedules than they were used to before the pandemic (WHO, 2020b). Longer working schedules in the assigned shifts, heavy workloads, and other psychosocial risks can lead to exhaustion, occupational burnout, elevated psychological discomfort, and negative mental health consequences [18,19]. While it has been observed that professionals working in the intensive care unit or emergency department in the battle against the pandemic are more emotionally exhausted than those working in the regular wards [20]. Teng et al. found that depression, anxiety, and fatigue are all frequent among frontline health workers [21].

A lack of supervised training on PPE, an insecure work environment, and poor working circumstances may lead to an elevated perception of risk to oneself and concern of transference to their family. This could lead to a significant decline in drive and unpleasant emotions like despair and guilt. As a result, organizations need to ensure guarantee the safety of HCWs and address their basic requirements as a top priority. It's also been discovered that peer support and supervisory assistance are linked to psychological well-being. The ability to converse with someone about their feelings or experiences, discuss the emotional and physical circumstances they face at work, and share their worries with co-workers can all alleviate feelings of isolation and stress. Resident physicians on the job should be motivated to interact with one another, and if necessary, support groups should be accessible via social networks. Additionally, individual psychological load appears to be linked to sentiments of occupational expertise during COVID-19-related duties. Providing appropriate pre-job training for those who will take a job on the front lines, outlining precise and reliable information about the disease, the threat of contagion, and the ability to treat oneself, as well as instituting systematic diagnostic and treatment procedures with specific guidelines, can enable alleviate stress and boost occupational esteem [22]. Peer support programs have been shown to improve resident physicians' psychological well-being and minimize the likelihood of burnout among clinicians [23]. A thriving trainee doctor peer support group will presumably foster a sense of connectedness and teamwork and give substance to experiences, reducing burnout and improving patient care in the long run [24].

Real-life clinical situations, simulation, academic lectures, small group sessions, journal clubs, and teaching sessions are all used in the education program of the residents. However, the COVID-19 pandemic, on the other hand, has hampered the residents' routine teaching and learning schedules. Considering that medical education is crucial to residents' career advancement, any disruption or alteration in their learning might negatively impact their success. During this time, online learning seemed to be the cornerstone of the learning process, since it could convey information while avoiding social contact, aiding in virus spread. Many residents believe that their study routine was disrupted during the epidemic; this adjustment has been linked to increased levels of stress and depression in students, according to these research studies [25].

Responders who perceived the pandemic had impacted their training and study schedule, who had lost service time, and who did not have the option to select whether or not to work with COVID-19 patients or who were pushed to, had considerably higher stress levels. Internal medicine, emergency medicine, and family medicine residents were rerouted to higher-need areas. Some essential rotations, such as pediatrics and anesthesia, were canceled entirely for these redeployments to occur. In addition, due to a transition to digital care and efforts to curb resident exposure, chances to learn and enhance technical knowledge came to a standstill. This is in line with the findings of a reported study, which found that junior residents were more likely to possess more severe mental health consequences like depression, anxiety, insomnia, and discomfort [11]. Lack of experience, insufficient preparation, or lack of training could all be plausible reasons for the observed effects. Hence, healthcare executives must create and implement support initiatives based on healthcare professionals' needs and wishes. Peer counseling with connectivity to a therapist, therapy sessions and counseling, and an online clinician support group, for example, received the most interest in a survey [26].

It is vital to assess the impact of limits in face-to-face work supervision and the merits and implications of giving training through alternative methods such as online platforms and their possible permanent integration into the curriculum. Similarly, in light of trainee concerns about their capacity to meet specific training needs and pass competency assessments, competence definitions and curricula need to be re-evaluated [25].

Limitations of the study

The survey's voluntary structure may have resulted in a selection bias, and the participants may not accurately reflect the actual population. A self-report questionnaire was employed to assess psychological symptoms, which did not concentrate on mental health specialists' diagnostic evaluation, to contact as many people as possible during this emergency situation, and reduce face-to-face interviews. We solely looked into the depression, anxiety, and stress levels of junior residents in this study. More research with a larger sample size incorporating social support and post-traumatic stress disorder assessment among HCW, on the other hand, would undoubtedly add to the literature.

## Conclusions

A pandemic necessitates swift organizational and healthcare personnel adaptability. It is, therefore, vital for organizations to consider factors that are independently associated with depression, anxiety, and stress which need to be taken into consideration to protect the Residents' Mental Well-being.

Supportive programs must be envisaged for health professionals engaged in an adverse event. Organizations must exploit the pandemic as a platform to devise focused initiatives to reduce significant stressors affecting healthcare employees' mental well-being under normal conditions. The downturn has taken a tremendous toll on healthcare professionals. Those in roles of institutional or sector leadership should seize this advantage to implement targeted initiatives to alleviate critical stressors that affect the mental health of healthcare professionals.
